# Analysis of Black Vomit in Yellow Fever

**Published:** 1855-01

**Authors:** 


*Analysis of Black Vomit in Yellow Fever.*

1. When black vomit is filtered, the colouring matter remains upon the filter, and the filtered fluid is colorless.

2. The specific gravity of the filtered fluid varies from 102 to 103.

3. The filtered fluid always reddens litmus.

4. Nitrate of silver when added to tho filtered fluid form a large quantity of white precipitate, which is insoluble in nitric acid, but soluble in ammonia; hence the fluid contains hydro-chloric acid.

5. Lime water, kali nitricum. and the other alkalies, form no other precipitate with the fluid, either when submitted to cold or heat.

6. A solution of the bi-chloride hidrarg. produces no apparent reaction with this filtered fluid.

7. Muriate and nitrate of baryta each prduces a slight turbulence when added to the filtered fluid.

8. Acetate of lead added to the filtered fluid causes a white flocky precipitate, which is insoluble in water, but soluble in acetic acid; hence mucous exists in the fluid.

9. A precipitate is formed by nitrate of silver in solution being added to the filtered fluid. If kali purum be added to this mixture, it deprives it of all acid; and then, afterwards, if sulphuric acid be added to this, a slight effervescence will ensue. Hence, we infer the existence of carbonic acid gas in the fluid.

*Coloring Matter of Black Vomit.*

1. It presents the appearance to the naked eye of blood globules which have been separated by chemical means from the blood.

2. When dried by artificial heat or the atmosphere, it resembles hemastosine procured from man's blood.

*Microscopic Analysis.*

A homogeneous fluid that contains—1. Blood globules entire. 2. Fragments of globules. 3 Mucus. 4. Epithelial cells. 5. Amorphus matter. 6. Granules sometimes. 7. Fat globules. 8. Torulæ and spores.—*New Orleans News & Hospital Gazette*, Sept. 1854.

